# Reaction graph kernels predict EC numbers of unknown enzymatic reactions in plant secondary metabolism

**DOI:** 10.1186/1471-2105-11-S1-S31

**Published:** 2010-01-18

**Authors:** Hiroto Saigo, Masahiro Hattori, Hisashi Kashima, Koji Tsuda

**Affiliations:** 1 Max Planck Institute for Informatics, Campus E1 4, 66123 Saarbrucken, Germany; 2 Bioinformatics Center, Institute for Chemical Research, Kyoto University, Uji, 611-0011 Kyoto, Japan; 3 Department of Mathematical Informatics, Graduate School of Information Science and Technology, The University of Tokyo, 7-3-1 Hongo, Bunkyo-ku, 113-8656 Tokyo, Japan; 4 AIST Computational Biology Research Center, 2-42 Aomi, Koto-ku, 135-0064 Tokyo, Japan

## Abstract

**Background:**

Understanding of secondary metabolic pathway in plant is essential for finding druggable candidate enzymes. However, there are many enzymes whose functions are not yet discovered in organism-specific metabolic pathways. Towards identifying the functions of those enzymes, assignment of EC numbers to the enzymatic reactions they catalyze plays a key role, since EC numbers represent the categorization of enzymes on one hand, and the categorization of enzymatic reactions on the other hand.

**Results:**

We propose reaction graph kernels for automatically assigning EC numbers to unknown enzymatic reactions in a metabolic network. Reaction graph kernels compute similarity between two chemical reactions considering the similarity of chemical compounds in reaction and their relationships. In computational experiments based on the KEGG/REACTION database, our method successfully predicted the first three digits of the EC number with 83% accuracy. We also exhaustively predicted missing EC numbers in plant's secondary metabolism pathway. The prediction results of reaction graph kernels on 36 unknown enzymatic reactions are compared with an expert's knowledge. Using the same data for evaluation, we compared our method with E-zyme, and showed its ability to assign more number of accurate EC numbers.

**Conclusion:**

Reaction graph kernels are a new metric for comparing enzymatic reactions.

## Background

**Figure 1:**
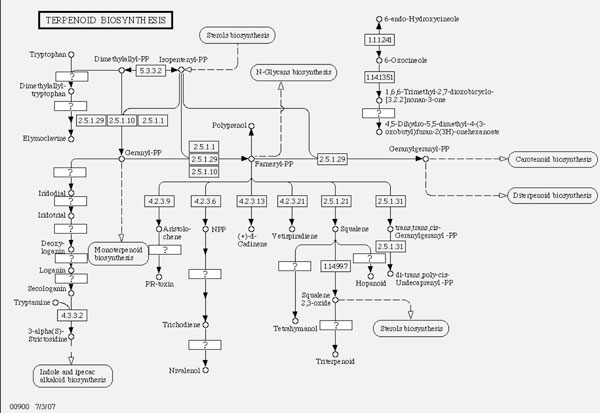
**Sample pathway**. A part of a terpenoid biosynthesis pathway extracted from KEGG/PATHWAY.

A metabolic network represents the transition or transformation of chemical compounds, where enzymes are represented as edges, and chemical compounds are represented as vertices. With the recent developments of pathway database: KEGG PATHWAY [1], much more information on chemical compounds and the roles of enzymes in biological systems has become available. In particular, many secondary metabolites found in plants are known to have roles in the defenses against pathogens, and have been attracting attention of researchers for more than a decade [2]. However, the organism-specific metabolic networks are not complete, and there are many "missing enzymes" whose existence are known but their functions are unknown. For identifying the characteristics of those missing enzymes, assignment of EC (Enzyme Classification) numbers to the enzymatic reactions plays a key role, since the EC number represents a hierarchical categorization of enzymes with respect to the enzymatic reactions they catalyze. So one can assign EC numbers to enzymatic reactions based on the knowledge from similar reactions first, then look up candidate enzymes in the same EC category. The process of assigning EC numbers is done manually by the Joint Commission on Biological Nomenclature (JCBN) of the International Union of Biochemistry and Molecular Biology (IUBMB) and the International Union of Pure and Applied Chemistry (IUPAC), however, this assignment process is so slow and many enzymes are still unannotated. For example, Figure 1 shows a part of a terpenoid biosynthesis pathway, but there are many enzymes whose EC numbers are not yet assigned (denoted as "?" in the boxes in the figure).

Fulfilling such missing EC numbers on a pathway can be casted as a multi-class classification problem given a pair of substrate and product as an input and the corresponding EC number as an output. Kotera et al. proposed an automatic EC number assignment system "E-zyme" for metabolic reactions [3]. Yamanishi et al. recently reformed the engine of E-zyme by introducing multi-layered matching and weighted majority voting [4]. However, E-zyme is still based on the detection of maximum common subgraphs between chemical compounds, so the shift of a large chemical group is not correctly detected [5]. Also, the E-zyme system is a rule-based method and does not allow approximate matching, which results in poor coverage. In many cases, E-zyme rejects a query because none of the rules matches [6].

In this paper, we propose to represent a metabolic reaction as a *reaction graph*, where each vertex corresponds to a chemical compound, and an edge between two chemical compounds corresponds to their relationships in reaction. A reaction graph is a 'graph of graphs', because each node contains a graph representing a chemical compound. To evaluate the similarity of two reaction graphs, we use marginalized graph kernels [7] in a recursive way. First, we compute graph kernels between every pair of chemical compounds and then use it as a node kernel for an upper-level graph kernel. In our experiment based on the KEGG/REACTION database, our reaction graph kernel in combination with kernel nearest neighbor showed 83% accuracy for classifying 4610 reactions into 124 classes. Furthermore, we exhaustively extracted missing enzymatic reactions in the plant's secondary metabolism in the KEGG database. Among the 56 reactions extracted, we could assign EC numbers to 36 reactions with the help of an expert from the KEGG team. The performance of our method is compared with E-zyme on this external validation set. Reaction graph kernel successfully assigned EC numbers to 22 EC classes, 14 EC subclasses and 12 EC subsubclasses. On the other hand, E-zyme could assign EC numbers to only 14 EC classes, 10 EC subclasses and 8 EC subsubclasses, due to its low coverage. The biochemical grounds for manual assignments are shown together with the individual prediction results of reaction graph kernels and E-zyme.

Data and supplementary information is available from http://www.mpi-inf.mpg.de/%7Ehiroto/RGKDATA/.

## Results and Discussion

### Reaction graph and reaction graph kernel

An example of metabolic chemical reaction is represented by(1)

Given such a chemical reaction, a task is to predict the EC number of the enzyme catalyzing the reaction. In this case, the enzyme is *secologanin synthase *(EC 1.3.3.9), which turns a substrate (*Loganin*) into a product (*Secologanin*) with *NADPH *as a cofactor. However, if the information on the enzyme is not available, we need to look up the entries in the database whose reactions are similar to the reaction of interest. A reasonable similarity metric is a key to solving this problem.

**Figure 2:**
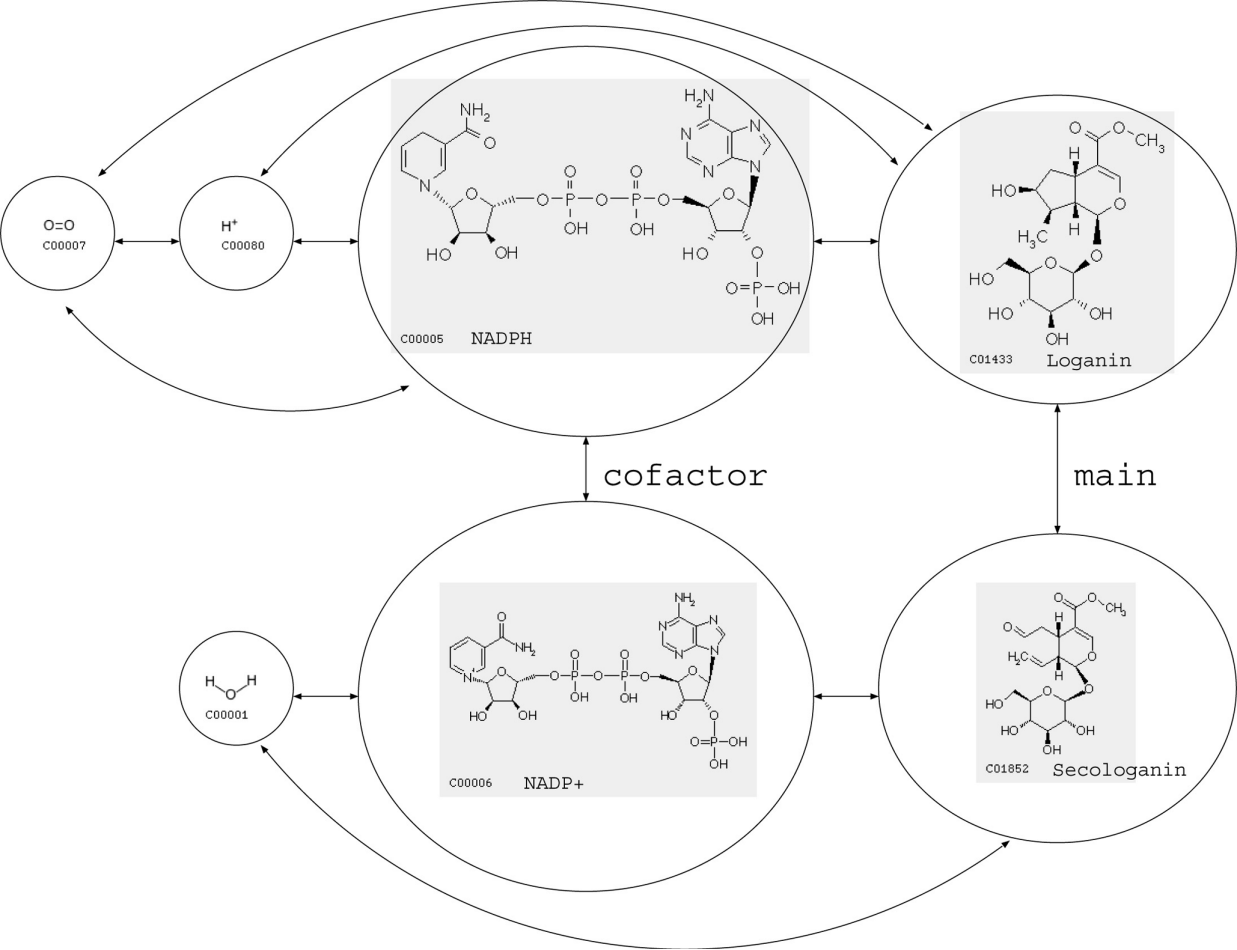
**Sample reaction graph (full-edge)**. The reaction graph for the reaction *Loganin *+ *NADPH *+ *H*^+ ^+ *Oxygen *<=> *Secologanin *+ *NADP*^+ ^+ *H*_2_*O*, which is catalyzed by *secologanin synthase (EC 1.3.3.9)*. Edges without labels are all 'group' edges in this reaction.

As a canonical representation of chemical reactions, we propose to represent metabolic reactions as *reaction graphs*. A reaction graph consists of vertices, which are compounds in a reaction, and edges which denote the relationships between compounds. The edge labels are chosen from either 'main', 'leave', 'cofactor', 'transferase' or 'ligase' based on the categorization in the KEGG/RPAIR database. We additionally introduced a 'group' edge which connects all the compounds on the substrate side of the reaction, and all the compounds on the product side of the reaction. An example of reaction graph corresponding to Equation (1) is presented in Figure 2.

To evaluate the similarity between two reaction graphs, we use random walk kernels [7] in a recursive way. We first compute all the pairwise similarities of the vertices (chemical compounds) using random walk kernels. Then the compound-wise similarities are used as the label matching probabilities for the upper-level graph kernel. The details of random walk kernels are described in the Methods section.

### Leave-one-out prediction of missing EC numbers

In order to evaluate the reaction graph kernels, we collected metabolic reactions from the KEGG/REACTION database. Following the pre-process used by [3], we did not use reactions which (i) do not have EC numbers, (ii) include chemical compounds whose structures are not available, (iv) have classes 97 and 99, (v) have only one reaction in the same subsubclass. This pre-processing resulted in 4, 610 reactions in 6 classes, 50 subclasses, and 124 subsubclasses.

In this experiment, we withheld one reaction from the database, and predicted its EC number using all the other reaction-enzyme pairs. For the prediction, we used the nearest neighbor approach based on the reaction graph kernels. For the calculation of the reaction graph kernels, we used Chemcpp (Available from http://chemcpp.sourceforge.net/) with the "non-tottering" option [8]. The random walk parameter of the lower-level and upper-level graph kernels were selected from {0.99, 0.8, 0.7, 0.6}, respectively, and 0.9 was used for both kernels, since it performed best in the experiments.

**Figure 3:**
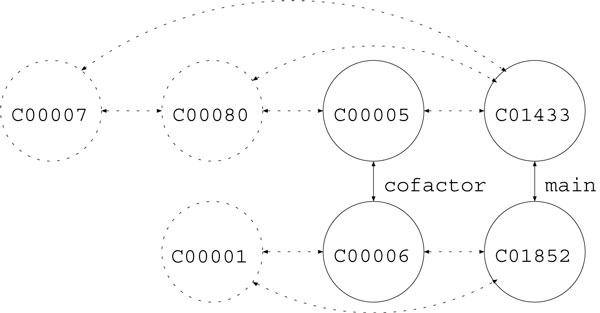
**Sample reaction graph (RPAIR)**.

**Figure 4:**
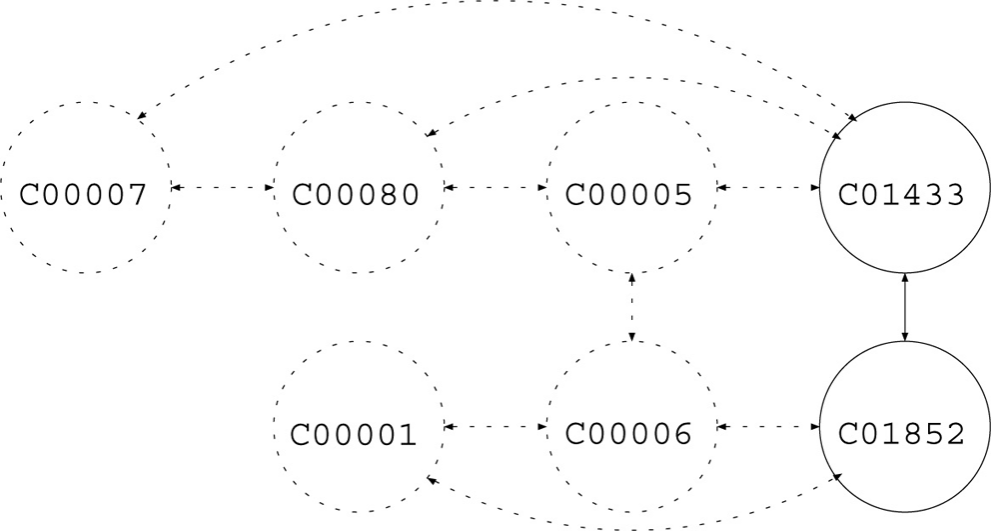
**Sample reaction graph (main-pair)**.

In reality, it is not often the case that the whole reaction graph of a query is known, so we considered degenerated settings, namely, RPAIR and main-pair. In the RPAIR setting, only reactant pairs are used, where the reactant pair information is obtained from KEGG/RPAIR database Figure 3. In the main-pair setting, only main-pairs are used for prediction Figure 4.

The leave-one-out accuracy is reported in Table 1. In the table, we denote the degenerated settings as RPAIR and main-pair, and the non-degenerated setting as full-edge. Clearly, predictions up to the second digit (EC subclass) and to the third digit (EC subsubclass) are more difficult. We did not test up to the fourth digit, since the last digit is often used just as a serial number [3]. We observed that additional edges in the reaction graphs help improve the classification performance. Notice that RPAIR corresponds to the same setting as that of E-zyme, but the use of full-edge turned out to be strongly advantageous in discriminating small changes in similar lower class reactions. According to [4], the E-zyme system has similar precision, as long as they provide an answer. However, its coverage is much lower than our method, as shown in the next subsection.

**Table 1:** Leave-one-out cross validation accuracy

	EC class	EC subclass	EC subsubclass
full-edge	94.8%	86.0%	82.5%
RPAIR	92.3%	81.4%	78.1%
main-pair	77.8%	69.8%	66.2%

### Predicting EC numbers of unannotated reactions in plant's secondary metabolism

In order to further evaluate the proposed method, we performed a blind test, where we tested only reactions whose EC numbers are not yet assigned in the secondary metabolism of plants. First we collected metabolic reactions from the KEGG "Biosynthesis of Secondary Metabolites - Reference pathway" data. From the resulting 56 reactions, we removed 20 reactions which are either non-enzymatic reactions or multi-step reactions whose systems are too complicated, based on an expert's judgement. Then we tested the E-zyme and reaction graph kernels on the remaining 36 reactions.

E-zyme returned answers to only 22 queries, and the coverage was only 61.1%. This is because E-zyme is a rule-based method, and can only match very similar reactions. Reaction graph kernels allow approximate matching, and returned answers for all the 36 reactions. The performance on the blind test is reported both for E-zyme and reaction graph kernels in Table 2. Reaction graph kernels could assign more number of correct EC numbers than E-zyme. However, E-zyme achieves slightly better accuracy. This is because E-zyme rejects queries which are too difficult to predict. It is worth noting that reaction graph kernels can also reject queries and achieves higher accuracy at the cost of coverage.

**Table 2:** Number of correct predictions and accuracy in top *k *candidates for 36 unknown reactions

		Coverage	EC main	EC sub	EC subsub
RGK	TOP1	100%	22 (61.1%)	14 (38.9%)	12 (33.3%)
	TOP3		56 (51.9%)	30 (27.8%)	24 (22.2%)
	TOP5		86 (47.8%)	37 (20.6%)	27 (15.0%)

E-zyme	TOP1	61.1%	14 (63.6%)	10 (45.5%)	8 (36.4%)
	TOP3		42 (63.6%)	24 (36.4%)	18 (27.3%)
	TOP5		57 (51.8%)	30 (27.3%)	24 (21.8%)

A list of newly annotated reactions is presented in Additional file 1, together with prediction results of E-zyme and reaction graph kernels (RGK). For reaction graph kernels, the Z-score (, where *x *is a raw score, and *μ *and *σ *are the mean and the standard deviation of the candidate scores) is calculated so that one can find a candidate with a saliently higher score than others. The biochemical grounds for the manual assignment of the EC numbers are presented in the "Comments" column. Since the enzyme nomenclature and the scheme of EC number classification had been published [9], we can infer the plausible EC numbers from given information of reaction formula. As can be seen in Additional file 1, some reactions progress in multiple steps and have several correct EC numbers. However, neither reaction graph kernels nor E-zyme considers such situations, which remains for future research.

## Conclusion

We proposed an alternative method for assigning EC numbers to unknown enzymatic reactions based on reaction graph kernels which measure similarity between reaction graphs. On a blind test predicting missing EC numbers in plant secondary metabolism pathway, we demonstrated that reaction graph kernels collected more number of accurate potential EC numbers than E-zyme.

## Methods

In this section, we introduce graph kernels that define similarity metrics between two labeled graphs.

### Random walk graph kernel

The key idea behind the random walk graph kernel is to use random walks on the given graphs to generate label sequences, and each graph is represented as a bag of label sequences from the random walks. The similarity of two graphs are defined as the number of common label sequences weighted by the probability of the corresponding walks (or more precisely, the probability of common label sequences being generated). The random walk graph kernel is a valid kernel, since it is interpreted as an inner product in the feature space spanned by the label sequences.

Let us assume that we want to define a similarity metric between two labeled graphs *G*_1 _= (*V*_1_, *E*_1_, *L*_1_(*V*_1_)) and *G*_2 _= (*V*_2_, *E*_2_, *L*_2_(*V*_2_)), where *V*_1 _and *V*_2 _are sets of vertices, *E*_1 _and *E*_2 _are sets of edges, and *L*_1 _and *L*_2 _are sets of labels of the vertices. Although our description assumes that the edges are not labeled we convert labeled edges to labeled vertices if the edges have labels. (Actually, we have bond labels in the lower-level graph kernel, and reaction labels in the upper-level graph kernel.) This conversion increases the number of vertices from |*V*_1_| + |*V*_2_| to |*V*_1_| + |*V*_2_| + |*E*_1_| + |*E*_2_| and doubles the number of edges.

We consider a joint random walk over the two graphs *G*_1 _and *G*_2 _to define our graph kernel. First, we define a random walk over one graph. Let ***u***_1_(*t*) be a |*V*_1_|-dimensional vector representing the probability distribution of the position of the random walk over the vertices in *G*_1 _at time *t*. The random walk starts with an initial distribution ***u***_1_(0). One possible choice of ***u***_1_(0) is the uniform distribution over *V*_1_. At each time step *t*, the random walk terminates with probability 1 - *λ*_1 _where 0 <*λ*_1 _< 1. The random walk proceeds with probability *λ*_1_, and moves to the next vertex by using a transition matrix ***T***_1_. The (*i, j*)-th element of ***T***_1 _indicates the probability of a transition from the *j*-th vertex to the *i*-th vertex in *G*_1_. One possible choice of *T*_1 _is the normalized adjacency matrix of *G*_1_. The dynamics of the random walks over *G*_1 _are given as

For example, when a random walk stops at time *t*, the probability distribution over *V*_1 _is represented as (1 - *λ*_1_) (*λ*_1_***T***_1_)^*t *^***u***_1_(0). A random walk over *G*_2 _is defined by using ***u***_2_(*t*), *λ*_2_, and ***T***_2 _defined accordingly. Since we want to compute the probability of two label sequences produced by the random walks matching, we consider the joint random walk using ***T***_1 _and ***T***_2 _over *G*_1 _and *G*_2_, respectively. Specifically, the joint distribution of the two random walks is given as ***U***(*t*) = ***u***_1_(*t*) ⊗ ***u***_2_(*t*)^⊤^, where ⊗ indicates the Kronecker product. Noting that the two random walks are independent of each other, the dynamics for the joint random walk is given as

Let ***M ***be a |*V*_1_| × |*V*_2_| vertex-wise kernel matrix. The values of ***M ***can take any values between zero and one according to the similarities between the labels. One simple choice of ***M ***is the Dirac kernel, where the (*i*_1_, *i*_2_)-th elements of ***M ***is 1 if the *i*_1_-th node in *V*_1 _and the *i*_2_-th node in *V*_2 _have an identical label, and is 0 otherwise. We will discuss the specific choice of ***M ***for our reaction kernel later. The dynamics of the "label matching" joint random walks are represented as(2)

where * is the Hadamard (element-wise) product, and ***V***(0) ≡ M * ***U***(0).

Then the matching probability (which is the graph kernel) is given as

where the (*i*_1_, *i*_2_)-th element of ***V ***is denoted by . Now our goal is reduced to computing the infinite sum . From Eq. (2), we have the relation

so we can use the fixed point iteration

used by [10] to update the current solution starting from  ← ***V***(0). The computational complexity of each update is *O*(|*E*_1_||*V*_2_| + |*E*_2_||*V*_1_| + |*V*_1_||*V*_2_|), where the first term and the second term are for applying ***T***_1 _and ***T***_2 _to , respectively. The third term is for the application of ***M***, so it can be replaced by the number of non-zero elements in ***M***. Therefore,  can be updated very efficiently if the graphs and ***M ***are sparse. The iteration is continued until convergence, but usually a few dozen steps are sufficient.

Our specific choices of ***M ***in our reaction graph kernel are as follows. For the upper-level reaction graph kernel, the elements of ***M ***for defining similarities among chemical compounds are replaced by the lower-level compound graph kernel, while we use the Dirac kernel for the elements of ***M ***for chemical reactions. In the lower-level compound graph kernel, we use the Dirac kernel for both bond similarities and atom similarities.

## Competing interests

The authors declare that they have no competing interests.

## Authors' contributions

H.K. drafted the Methods section. H.S. and K.T. drafted the rest of the manuscript. H.S. performed computational experiments using reaction graph kernels. M.H. evaluated E-zyme and annotated reactions in plant's secondary metabolism.

## Supplementary Material

Additional File 1 **Results in plant secondary metabolism pathway**. A list of newly annotated reactions in plant secondary metabolism in xls format. "NA" in the E-zyme column means that no answer was available for that query. CXXXX is a KEGG compound ID. Correctly assigned EC numbers are highlighted in bold fonts. Click here for file
